# Exercise intolerance and developmental delay associated with a novel mitochondrial ND5 mutation

**DOI:** 10.1038/srep10480

**Published:** 2015-05-27

**Authors:** Hezhi Fang, Hao Shi, Xiyuan Li, Dayan Sun, Fengjie Li, Bin Li, Yuan Ding, Yanyan Ma, Yupeng Liu, Yao Zhang, Lijun Shen, Yidong Bai, Yanling Yang, Jianxin Lu

**Affiliations:** 1 Key Laboratory of Laboratory Medicine, Ministry of Education, Zhejiang Provincial Key Laboratory of Medical Genetics, College of Laboratory Medicine and Life sciences, Wenzhou Medical University, Wenzhou 325035, Zhejiang, China; 2 Department of Pediatrics, Peking University First Hospital, Beijing 100034, China; 3 Department of Cellular and Structural Biology, University of Texas Health Science Center at San Antonio, San Antonio, TX 78229, USA

## Abstract

The aim of this study was to evaluate the contribution of mitochondrial DNA (mtDNA) mutations in oxidative phosphorylation (OXPHOS) deficiency. The complete mitochondrial genomes of 41 families with OXPHOS deficiency were screened for mutations. Mitochondrial functional analysis was then performed in primary and cybrid cells containing candidate mutations identified during the screening. A novel mitochondrial NADH dehydrogenase 5 (ND5) m.12955A > G mutation was identified in a patient with exercise intolerance and developmental delay. A biochemical analysis revealed deficiencies in the activity of complex I (NADH:quinone oxidoreductase) and IV (cytochrome c oxidase) of this patient. Defects in complexes I and IV were confirmed in transmitochondrial cybrid cells containing the m.12955A > G mutation, suggesting that this mutation impairs complex I assembly, resulting in reduced stability of complex IV. Further functional investigations revealed that mitochondria with the m.12955A > G mutation exhibited lower OXPHOS coupling respiration and adenosine triphosphate (ATP) generation. In addition, the cytotoxic effects, determined as reactive oxygen species (ROS) and lactate levels in the present study, increased in the cells carrying a higher m.12955A > G mutant load. In conclusion, we identified m.12955A > G as a mitochondrial disease-related mutation. Therefore, screening of m.12955A > G is advised for the diagnosis of patients with mitochondrial disease.

Mitochondria play a central role in a variety of cellular processes, including oxidative phosphorylation (OXPHOS), cell signaling, calcium buffering, and apoptosis^1^. There are approximately 1000 proteins in the mitochondria, only 13 of which are encoded by mitochondrial DNA (mtDNA). Most mitochondrial diseases are caused by nuclear-encoded DNA mutations and follow Mendel’s laws of inheritance^2^. However, many fatal pathological conditions such as Leigh syndrome, leukoencephalopathy, and some degenerative diseases such as cancer, Leber hereditary optic neuropathy (LHON), and mitochondrial myopathy, encephalopathy, lactic acidosis, and stroke-like episodes (MELAS) are associated with mtDNA mutations^2^.

Mitochondrial respiratory complex I (NADH:quinone oxidoreductase), which is the first and largest enzyme in OXPHOS, is the major electron entry point to the mitochondrial respiratory chain. A long-term survey of mitochondrial respiratory chain disorders in Australian children showed that OXPHOS disorders were frequently observed in newborns, with an estimated incidence of 1 in 5,000 and complex I defects accounted for approximately 25–35%^3^,^4^ of these cases. Complex I is composed of 38 nuclear subunits and seven mtDNA encoded subunits, which are assembled in the inner mitochondrial membrane to form an L-shaped structure. In mammalian cells, complex I consists of 14 core subunits for the assembly of the complex I scaffold motif and 31 additional supernumerary subunits of unknown function^5^,^6^. In vertebrates, all seven mtDNA encoded hydrophobic subunits [mitochondrially encoded NADH dehydrogenase 1–6 and 4L (ND1-ND6 and ND4L)] are core subunits of complex I and form the major complex I membrane arm. Mutations in NADH dehydrogenase (ND) subunits can disrupt either complex I assembly or enzyme activity^7^. Recent studies have shown that approximately 20% of cases of isolated complex I deficiency were caused by mtDNA mutations in mitochondrially encoded NADH dehydrogenase (MTND) genes^8^. In particular, mutations in ND subunits were associated with LHON, MELAS, and Leigh Syndrome^9^. New pathogenic MTND mutations are being frequently identified, but the functional consequences of these mutations have not been confirmed^10^,^11^. Elucidation of the functional effects of pathogenic mtDNA mutations will aid in the genetic diagnosis of patients with complex I deficiency. However, the identification of new pathogenic mtDNA mutations is difficult due to the diversity of mtDNA polymorphisms and the clinical heterogeneity of disease.

In this study, we screened the whole mitochondrial genome in a large cohort of 41 pediatric patients with biochemical manifestations of complex I deficiency. Patients with recurrent mutations such as m.3243A > G, m.8993T > G/C and m.8344A > G were ruled out of the study before the sequence screening. The reported pathogenic mtDNA mutations m.3697G > A and m.14487T > C were identified in two patients with MELAS and Leigh syndrome, respectively^12^,^13^. Furthermore, we identified one patient with exercise intolerance and developmental delay harboring a novel pathogenic mutation m.12955A > G [p. Asn(N)207Asp(D)]. To further confirm the pathogenic mechanism of the m.12955A > G mutation, a series of cytoplasmic hybrid (cybrid) cell lines with different loads of the m.12955A > G mutation were constructed by transferring mitochondria from the patient into mtDNA-less ρ0 human osteosarcoma 143B cells. The effects of the m.12955A > G mutation on OXPHOS deficiency was analyzed by assessing mitochondrial respiration, adenosine triphosphate (ATP) content, reactive oxygen species (ROS) levels, and lactate acid levels in addition to complex I assembly and activity.

## Results

### Clinical data

Samples from 41 patients diagnosed with OXPHOS deficiency and their maternal family members were obtained from Peking University First Hospital (China). Informed consent was obtained from all subjects under protocols approved by the Ethical Committee of the Peking University First Hospital. All experimental methods were carried out in accordance with approved guidelines of Peking University First Hospital. The patient (female, aged 9 years at the time of this study) identified with the novel mtDNA 12955A > G mutation, was born in a non-consanguineous Chinese family and presented with clinical manifestations of exercise intolerance and developmental delay. The child was initially investigated for muscle weakness, malnutrition and developmental and psychomotor retardation at the age of 6 months. She was referred to our hospital at the age of 2 years and 3 months owing to her inability to walk. The patient showed progressive deterioration in terms of muscle pain, severe fatigue, and dizziness, which were exacerbated by a short period of exercise. Physical examination indicated that the patient had limb hypotonia, muscle relaxation, knee tendon reflex hyperfunction with normal ankle clonus and Babinski sign, as well as reduced growth [weight: 22 kg (normal 24.1–35.3 kg)]; height: 123 cm (normal 125.7–138.7 cm). Magnetic resonance imaging of the brain revealed no pathological alterations. Cerebrospinal fluid (CSF) and blood lactate levels were 1.3 mmol/L (normal range, 1.0–2.8 mmol/L) and 3.9 mmol/L (normal range, 0.5–2 mmol/L), respectively. The lactate/pyruvate ratio in the CSF was 15 but reached 58 in blood (normal range <20), suggesting a respiratory chain dysfunction in this patient. Following treatment with a combination of L-carnitine (1 g/day), calcium folinate (15 mg/day), arginine (1 g/day), vitamin B1 [30 mg twice daily (b.i.d.)], vitamin B2 (5 mg b.i.d.), vitamin C (100 mg/day), vitamin E (50 mg b.i.d.) and coenzyme Q10 (10 mg b.i.d.) at 2 years and 3 months of age, the patient’s symptoms improved significantly and she was able to walk unaided after 3 months of treatment.

### Biochemical analysis

The enzyme activity of complex V (ATP synthase) and four mitochondrial respiratory chain complexes (MRCs) were measured in the lymphoblasts of the patient, as previously described^14^. The activity of each enzyme was normalized against that of citrate synthase, a mitochondrial matrix marker enzyme (Table 1). Based on the criteria of OXPHOS enzyme activity from 100 healthy children, defective complexes I and IV were identified in the patient (Table 1).

### Identification of pathogenic mutations

**Figure 1:**
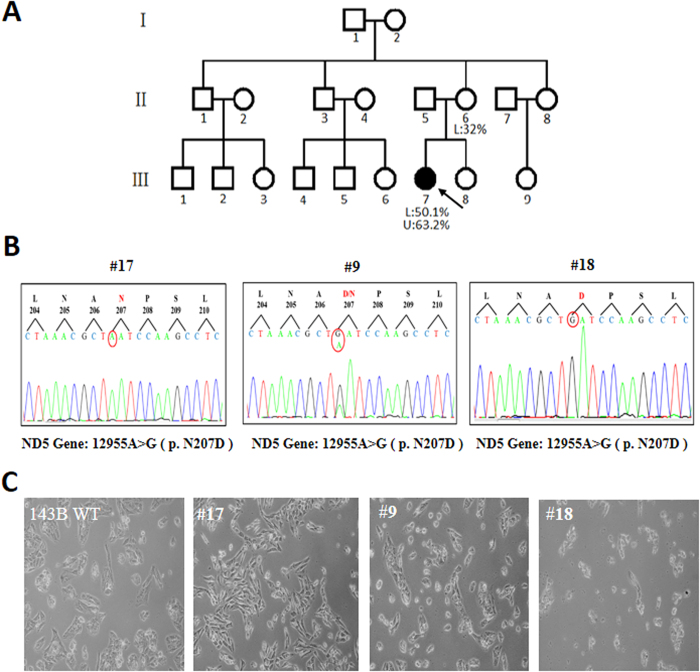
Family tree and cell models of m.12955A>G. (**A**) The proband is indicated by an arrow; mutant loads of lymphocytes (L) and urinary epithelium (U) were determined by real-time PCR. (**B**) Sequence diagrams of clone #9, #17, and #18 cells, N: Asn, D: Asp. (**C**) Morphology of cells after culturing for 24 h; 143B wild type (WT): cells without mtDNA mutation.

To identify the pathogenic role of these MRCs deficiencies, the whole mitochondrial genome of the patient and her maternal relatives were subjected to Sanger sequencing. The patient was found to be carrying an unreported heteroplasmic mutation, m.12955A > G (p.N207D). The mutant load was quantified by subjecting the blood and urine samples from the patient and a blood sample from her mother to real-time polymerase chain reaction (qPCR). As shown in Fig. 1A, the mutant load was higher in the blood of the patient compared with that in the blood of her mother (50.1% vs. 32%, respectively). In the urinary epithelium of the patient, the mutant load of m.12955A > G was even higher (63.2%). Since blood and urinary epithelium always contain lower mutant loads than do other tissues, we believed that a higher mutant load of m.12955A > G would be found in other tissues such as brain and muscle from the patient^15^,^16^. Unfortunately, we were unable to analyze the mutant loads in muscle and fibroblasts from the patient. In addition, the patient’s parents declined further investigations since our diagnosis of mild clinical manifestation was similar to a previous report by Downham *et al.*^17^. In addition, no complex IV-related mtDNA mutations were detected, although a decline of complex IV activity was identified in this patient (Table 2). Since the stability and activity of some MRCs are interdependent, it is possible that the complex I deficiency caused by m.12955A > G may decrease the assembly/stability of complex IV^18^,^19^. Furthermore, the possibility of a rare polymorphism at 12955 was excluded by screening for the mutation in 161 control subjects.

The features of m.12955A > G were further analyzed to evaluate its pathogenicity. A protein structure model of ND5 revealed that residue 207 is located in the third hydrophilic extramembrane loop (http://www.uniprot.org/uniprot/P03915). An N to D substitution introduces a negative charge at residue 207 in the extramembrane loop, thereby changing the electrostatic forces, which are important for protein-protein interactions^20^. Together, these findings indicated the pathogenic potential of the m.12955A > G mutation.

### Construction of cybrid cell lines containing different m.12955A>G mutant loads

To understand the pathogenic role of m.12955A > G fully, we generated a series of cybrid cell lines containing different loads of the mutation. Briefly, platelets from the patient were fused with mtDNA-less ρ0 human osteosarcoma 143B cells, and 20 single cybrid clones were selected by culturing the fusion mixtures in medium without uridine and sodium pyruvate for 15 days. Clone #9, #17, and #18 cells with mutant loads of 65.21%, 21.76% and 98.14%, respectively, were selected by real-time PCR for further analysis. However, owing to the poor sensitivity of Sanger sequencing in detecting of heteroplasmic mutations at mutant loads below 20%, the mutation was not detected in the sequencing of clone #17 (Fig. 1B).

Analysis of the cell morphology of the selected clones revealed that increased loads of the m.12955A > G mutation were associated with decreased cell viability (Fig. 1C). Cells with 20% of the m.12955A > G mutation had a smooth cell membrane and an elongated cell shape, which were comparable with that observed in wild type 143B cells, while cells with 65% of the heteroplasmic m.12955A > G mutation were smaller. Furthermore, cells with 98% of heteroplasmic m.12955A > G cells were aggregated and showed reduced viability (Fig. 1C). This result indicates that an m.12955A > G mutation affects cell viability in a mutant load-dependent manner. Due to the similar morphology between clone #17 cells and wild type 143B cells, and the observation that the m.12955A > G mutant load in the mother of the patient was approximately 30% but without the clinical phenotype, clone #17 cells were used as a control for clone #9 and #18 cells.

### Blue native PAGE analysis of respiratory complex assembly

**Figure 2:**
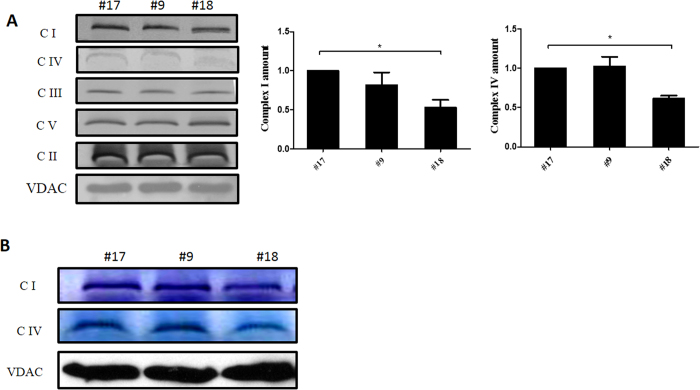
Respiratory complex assembly and in-gel activity assay. (**A**) Whole cells were solubilized with n-dodecyl-β-D-maltoside (DDM) and then subjected to BN-PAGE/immunoblot analysis. Complexes I, II, III, IV, and V were immunoblotted with anti-Grim19, SDHA, core2, COX IV, and ATP-5A antibodies, respectively; β-VDAC was used as internal control. (**B**) Mitochondria were solubilized with DDM. In-gel activity assays of complexes I and IV were performed following BN-PAGE analysis; the VDAC protein was used as a loading control. All BN-PAGEs were run under the same conditions (5 mA per gel for 1 h and followed by electrophoresis at 200 V for 2 h). Full-length blots are presented in Supplementary Figs. 1 and 2. Error bars, ±SD *, *P* < 0.05.

Intact complex I was analyzed by blue native polyacrylamide gel electrophoresis (BN-PAGE) to evaluate whether the m.12955A > G mutation impairs the assembly of complex I. Mitochondria isolated from the cells of clone #9, #17, and #18 were separated by BN-PAGE to assay for in-gel complex I activity and immunoblotting analysis of complex I with an antibody against NADH dehydrogenase (ubiquinone) 1 alpha subcomplex 13 (NDUFA13). As shown in Fig. 2A, cells of clone #18 cells had less intact complex I but more subcomplex I just below the position of complex I, compared to that observed in the cells of clone #9 and #17. However, complex I assembly in clone #9 was not affected when compared with clone #17 cells, suggesting that complex I assembly was impaired only by high m.12955A > G mutant loads. Moreover, a considerable decrease in complex IV assembly was found in clone #18 cells compared with clone #17 cells, while assembly of complex IV was normal in clone #9 cells. This indicates that the decrease in complex I assembly in clone #9 cells is not sufficient to affect complex IV assembly. Additionally, the assembly of complex II (succinate dehydrogenase), complex III (ubiquinol-cytochrome c reductase), and complex V were not affected in either clone #9 or #18 cells, compared with clone #17 cells (Fig. 2A). In-gel activity assays confirmed that the nearly homoplasmic mutant cybrid cells of clone #18 cells exhibited a significantly reduced complex I and IV activity relative to that of clone #17 cells (Fig. 2B). Together, our results indicate that a high m.12955A > G mutant load can impair complex I assembly, which may further affect the stability of complex IV.

### Mitochondrial functional analysis

**Figure 3:**
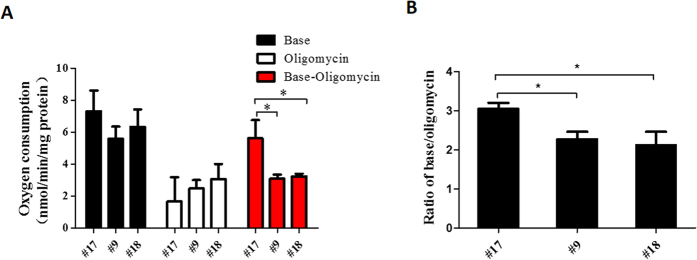
Respiration assay of cells containing the m.12955A>G mutation. (**A**) Mitochondrial respiratory capacities were determined in clone #9, #17, and #18 cells. Oligomycin (2.5 μg/ml) was added for the measurement of uncoupled mitochondrial respiration. OXPHOS coupling respiration was calculated by subtracting the uncoupled component value from the total endogenous respiration value. (**B**) Respiratory coupling efficiencies were presented as the ratio of basal oxygen consumption to the oxygen consumption with oligomycin. Error bars, ±SD. *, *P* < 0.05;

The endogenous respiration of intact cells was first measured to clarify the role of increased m.12955A > G mutant loads on mitochondrial function. As shown in Fig. 3A, although no significant decrease in base respiration was detected in intact cells, clone #9 and #18 cells showed a trend towards decreased respiratory function. Furthermore, coupled and uncoupled mitochondrial respiration among clone #9, #17, and #18 cells was measured in the presence of oligomycin, which completely blocks the ATPase proton channel. Clone #17 cells exhibited higher coupled OXPHOS respiration and respiratory coupling efficiency compared with clone #9 and #18 cells (Fig. 3A and B). Since only clone #18 cells exhibited a significantly reduced complex I [NADH:nitrotetrazolium blue (NTB) oxidoreductase] activity relative to that of clone #17 cells in in-gel activity assay (Fig. 2B), complex I activity of clone #9 cells was further measured as NADH:ubiquinone oxidoreductase using spectrophotometry-based complex I enzyme activity assay. As shown in supplementary Fig. 3, clone #9 cells exhibited a significantly reduced complex I (NADH:ubiquinone oxidoreductase) activity compared with control #17 cells. These observations suggested that the m.12955A > G mutation in ND5 impaired mitochondrial function by decreasing both the total mitochondrial respiration and the respiratory coupling efficiency.

**Figure 4:**
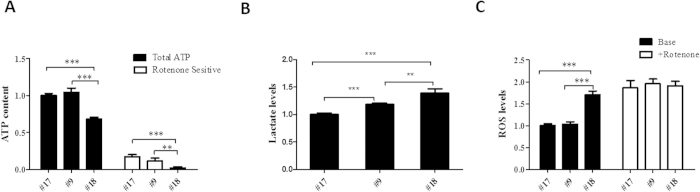
Mitochondrial function and cytotoxicity. (**A**) Total ATP content was measured in clone #9, #17 and #18 cells; the rotenone-resistant ATP content was determined in cells treated with 200 nM rotenone for 24 h. The rotenone-sensitive (RS) ATP production was calculated by subtracting the rotenone-resistant component from the total ATP content. (**B**) Extracellular lactate levels were measured in the medium after 48 h in culture. (**C**) Mitochondrial ROS were determined in clone #9, #17, and #18 cells; complex I-dependent ROS levels were measured in cells exposed to 1 μM rotenone for 1 h. The values for ATP, lactate, and ROS were normalized by protein concentration. Error bars, ±SD. *, *P* < 0.01; **, *P* < 0.001.

To further confirm the effect of m.12955A > G on mitochondrial function, ATP synthesis was determined in boiled cells by luminometry. As shown in Figure 4A, although the total ATP content in cells that were 65% heteroplasmic (clone #9) were not significant different with those in cells that were 21% heteroplasmic (clone #17), clone #9 cells showed a trend towards decreased ATP generation. Furthermore, the ATP generation in cells that were 98% heteroplasmic (clone #18) was much lower, suggesting a positive correlation between mitochondrial dysfunction and m.12955A > G mutant load. After the addition of rotenone, the ATP generation by respiratory complex I decreased with an increasing m.12955A > G mutant load (Fig. 4A). Thus, our findings further confirm that m.12955A > G impairs mitochondrial function by affecting complex I.

To test the toxicity of the m.12955A > G mutation, extracellular lactate acid and mitochondrial ROS levels were measured in all three cell lines. As shown in Fig. 4B, lactate acid generation increased with the mutant load of m.12955A > G. Furthermore, a significant increase in mitochondrial ROS was detected in clone #18 cells compared with that in clone #17 (Fig. 4C). After treatment with the complex I-related ROS-inducer rotenone for 1 h, both clone #9 and #17 cells showed a dramatic increase (approximately 1 fold for both) in ROS generation, while the increase in clone #18 cells was slight (approximately 20%). Interestingly, the ROS levels induced by rotenone in clone #9 and #17 cells were comparable with those in clone #18 cells with or without rotenone. This indicated that the high ROS levels in clone #18 cells were caused by the impairment of complex I.

To clearly understand the implications of m.12955A > G in mitochondrial dysfunction, the effect of the mutation in mitochondrial function was also determined by comparing clone #9, #17, and #18 with 143B wild type cells. As shown in Supplementary Fig. 4A, we found that both clone #9 and #18 cells presented a significant decrease of complex I-related ATP generation relative to that in 143B wild type cells. Clone #17 cells showed a mild decrease of complex I related ATP generation when compared to 143B wild type cells. In Supplementary Fig. 4B, the toxicity test for the m.12955A > G mutation showed that clone #9, #17, and #18 cells exhibited higher mitochondrial ROS generation than did 143B wild type cells. After the addition of rotenone, the mitochondrial ROS levels were more or less the same in the four cell lines, suggesting that the m.12955A > G mutation increases mitochondrial ROS generation through the impairment of complex I. Taken together, our results confirm that the mutation of m.12955A > G in complex I causes disease.

## Discussion

In the present study, we have identified a novel missense m.12955A > G mutation in the mitochondrial *ND5* gene from a patient diagnosed with exercise intolerance and developmental delay. To evaluate the casual role of the m.12955A > G mutation in disease pathogenesis, a detailed analysis of the mutation at the clinical, genetic, and molecular levels was performed. As shown in the case report, the patient with a high level of the 12955A > G mutation exhibited a metabolic defect that was partially alleviated by the administration of a combination of drugs for the treatment of OXPHOS deficiency. Together with the fact that the m.12955A > G mutant load in the patient was much higher than that in her mother, this observation suggested a possible correlation with disease occurrence. Unfortunately, we were unable to analyze the mutant loads in muscle and fibroblast cells from the patient as the patient’s parents did not allow further investigations. However, the heteroplasmy of mtDNA mutations are usually higher in muscle and fibroblast cells than in the blood and urinary epithelium^21^. Genetically, m.12955A > G was the only variation in the patient that was not listed in the mtDNA polymorphism databases from MITOMAP (http://www.mitomap.org), mtDB (http://www.genpat.uu.se/mtDB/) and mtSNP (http://mtsnp.tmig.or.jp). Moreover, we found that m.12955A > G was not detected in 161 Chinese control subjects. Thus, we speculate a possible pathogenic role of 12955A > G in human diseases.

At the molecular level, the activity of mitochondrial complexes I and IV was decreased in cybrid cells containing 98% of the m.12955A > G mutation (clone #18), which was consistent with the biochemical phenotype of the patient. As a result, a mutant load of 98% of m.12955A > G resulted in down-regulated mitochondrial biogenesis by decreasing OXPHOS-coupled mitochondrial respiration and OXPHOS-related ATP generation. Thus, tissues such as neurons and muscle, with a high OXPHOS demand, will be primarily affected. On the other hand, the mutant load of 98% m.12955A > G increased cytotoxicity by elevating both mitochondrial ROS and lactate acid levels. Although the intact respiratory complex levels and ROS generation were not affected in the 65% heteroplasmic 12955A > G cells, the decrease in respiratory coupling efficiency was comparable with that of the homoplasmic mutant (98%) cells. Functional studies further confirmed a trend towards lower ATP generation and a significant increase in lactate acid levels in the cells with 65% heteroplasmic of m.12955A > G compared with that of the cells with 21% heteroplasmic of m.12955A > G. All these above results strongly support the hypothesis that the m.12955A > G mutation is causative and that the severity of the disease is largely dependent on the mutant load.

ND5 is one of 14 essential subunits that constitute the backbone of respiratory complex I^5^. Deficiencies in ND1, ND2, ND4, and ND6 are proposed to disrupt complex I assembly, while defects in ND3 and ND5 have modest effects on complex I assembly but marked effects on enzyme activity^9^,^22^,^23^. Previously, we demonstrated that mitochondrial respiration is tightly controlled by ND5 by using a cell model with a nonsense ND5 mutation^24^. Here, only a mild decrease in endogenous oxygen consumption was observed in cells containing both 65% and 98% mutant loads of m.12955A > G compared with endogenous oxygen consumption in cells containing 21% mutant loads of m.12955A > G. However, the OXPHOS-coupled respiration was dramatically decreased in these two cybrid cell lines. Determination of the crystal structure of the bacterial complex I indicate that NADH-quinone oxidoreductase subunit 12 (Nqo12), a homolog of human ND5, is composed of a putative proton translocation channel and the residue of 207 is close to the proton-pumping channel and ubiquinol-binding site^6^. Thus, it can be speculated that the 12955 (p.N207D) mutation is more likely to affect proton pumping and ubiquinol binding directly. This speculation was confirmed in our results that the NADH:ubiquinone oxidoreductase rather than NADH:NTB oxidoreductase of complex I was decreased in clone #9 cells compared with clone #17 cells. In addition, the level of fully assembled complex I is decreased in cells carrying the homoplasmic m.12955A > G mutation, which is consistent with previous reports that ND5 is involved in complex I assembly^25^. Furthermore, we found a decreased assembly of complex IV in cells with 98% of m.12955A > G mutation, but not in cells with 65% m.12955A > G mutation, which have relatively normal complex I assembly. Thus, it is more likely that the stability of complex IV is partially dependent on its assembly into a supercomplex containing complex I. A similar theory has been suggested by others and us. Indeed, in ours previous report, was found that complex IV was essential for the assembly and stability of complex I^19^.

Being the largest subunit of 13 mtDNA-encoded proteins, mutations in the ND5 subunit are frequently observed in patients with mitochondrial defects^26^. ND5 mutations have been reported in many types of mitochondrial diseases with a broad spectrum of clinical phenotypes ranging from mild to severe^17^. Diseases such as LHON^27^, adult encephalopathy^11^, MELAS^28^, myoclonic epilepsy with ragged red fibers (MERRF)^29^, and Leigh syndrome^30^ have been reported in patients carrying ND5 mutations. Clearly, the etiology of diseases related to ND5 mutations covers a broad spectrum and exact correlations between the site of mutation and the clinical outcome have yet to be defined^17^. The ND5 missense mutation 13371T > C, which is located in the third extramembranous domain facing the intermembrane space, has been identified in relation to muscular exercise intolerance but was not associated with other neurological abnormalities^17^. Interestingly, the patient with the 12955A > G mutation exhibited a similar clinical phenotype to that of the patient carrying the 13371T > C mutation, the only difference being that 12955A > G is located in the third extramembranous domain facing the mitochondrial matrix, while 13371T > C is located in the ninth transmembrane helix (location determined using http://www.uniprot.org/).

In summary, we report 12955A > G as a new missense mutation in the ND5 gene that is associated with exercise intolerance and developmental delay. Investigations of the underlying mechanism revealed that this mutation impaired mitochondrial oxidative phosphorylation which further increased cytotoxicity in a mutant load-dependent manner.

## Materials and Methods

### Mitochondrial respiratory complex enzymatic activity assay

Mitochondrial respiratory complex enzyme activities were measured in patient lymphocytes and cultured cells as described previously^14^,^31^.

### Cell cultures

Transmitochondrial cybrids were obtained by fusion of mtDNA-less ρ0 human osteosarcoma 143B cells with platelets derived from the patient and her mother as described previously^32^. The transformant clones were cultured in high glucose Dulbecco’s modified Eagle’s medium (DMEM; HyClone, Waltham, USA) containing 10% cosmic calf serum (Gibco, Carlsbad, USA).

### mtDNA analysis

Genomic DNA was extracted using a sodium dodecyl sulfate (SDS) lysis protocol as described previously^33^. The entire mtDNA genome was Sanger sequenced using 24 previously reported pairs of mtDNA primers^34^. To quantify mutant loads, an allele specific amplification based real-time PCR method was used to determine the percentage of 12955G in total mtDNA. Real-time PCR reactions were performed on a Step-One plus Real-Time PCR system (Applied Biosystems, Foster City, USA) using the SYBR® Green qPCR Master Mix (Takara, Dalian, China). The sequences of the allele specific primers were as follows: mtDNA 12955A: forward, 5’-CAAATAGCCCTTCTAAACGCTA-3’; mtDNA 12955G: forward, 5’-CAAATAGCCCTTCTAAACGCTG-3’; reverse primer for mtDNA 12955A and mtDNA 12955G, 5’-CGCTGAGCCAGTCAGTGT-3’. The PCR amplification efficiencies of these primers were between 90% and 110%. The mutant load of 12955G was calculated using the following equation: Percentage of 12955G = 2^(Ct12955A-Ct12955G)^/(1 + 2^(Ct12955A-Ct12955G)^) × 100%. For the analysis of m.12955A > G mutations in healthy individuals, the primers listed below were used to sequence the ND5 gene: forward, 5’-AAACAACCCAGCTCTCCCTAA-3’; reverse, 5’- TCGATGATGTGGTCTTTGGA -3’.

### Oxygen consumption

Endogenous oxygen consumption by intact cells was determined using a Clark-type oxygen electrode (Hansatech, Norfolk, United kingdom) as described previously^35^. After recording the basal respiration, oligomycin (2.5 μg/ml) (Sigma, St. Louis, USA) was added to measure the un-coupling respiration of the cells.

### ATP measurements

ATP was measured using an ATP measurement kit (Molecular Probes, Carlsbad, USA) according to the manufacturer’s instructions. Briefly, cells were grown in 6-well plates to approximately 80% confluence. Approximately 1 × 10^6^ cells were washed with cold phosphate buffered saline (PBS) buffer, and then boiled in 100 μl Boiling Buffer (100 mM Tris, 4 mM EDTA, adjusted to pH 7.75 with acetic acid) for 90 s. Supernatants were retrieved by centrifugation at 10,000 × g for 1 min. ATP contents were determined by measuring the luminescence of supernatants mixed with Luciferase Assay buffer using a Varioskan™ Flash Multimode Reader (Thermo Scientific, Waltham, USA). ATP luminescence was normalized by protein concentration. To measure the generation of respiratory complex I-related ATP, a parallel set of cells was incubated with 200 nM rotenone (Sigma, St. Louis, USA) for 24 h before measuring the ATP.

### Lactate and ROS measurement

The extracellular lactate level was measured using a fluorimetric-based lactate assay kit (Amplite, Foster city, USA) according to the manufacturer’s instructions. Briefly, culture medium was filtered through a 10 kDa molecular weight (MW) spin filter (Millipore, Darmstadt, Germany), and fluorescence was record using a Varioskan™ Flash Multimode Reader (Thermo Scientific, Waltham, USA).

Mitochondrial ROS were measured according to published protocols^36^. Briefly, cells were washed in Hank’s buffered salt solution (HBSS), resuspended in HBSS containing 5 μM Mito SOX (Molecular Probes, Carlsbad, USA) and incubated at 37°C for 5 min. Cells were then washed (twice) with HBSS and fluorescence was recorded using a Varioskan™ Flash Multimode Reader (Thermo Scientific, Waltham, USA). To measure ROS levels in the presence of rotenone, cells were incubated with 1000 nM rotenone for 1 h.

### Mitochondrial protein preparation, blue native PAGE, and in-gel activity assay

Mitochondria from cultured cells were isolated as previously described^37^. Mitochondrial membrane proteins were isolated from whole cells or mitochondria with n-dodecyl-β-D-maltoside (DDM, Sigma, St. Louis, USA) at the required ratio^38^. Protein (60 μg) containing 0.5% Blue G-250 (Sigma, St. Louis, USA) and 5% glycerol were separated by BN-PAGE (3–11% gel) as previously described^38^. For complex I in-gel activity assays, gels were soaked in assay buffer [25 mg nitrotetrazolium blue (NTB) (Sigma, St. Louis, USA) and 10 μl 1 mg/ml reduced nicotinamide adenine dinucleotide (NADH) (Sigma, St. Louis, USA) in 10 ml 5 mM Tris/HCL (pH 7.4)] for approximately 1 h. For complex IV assays, gels were soaked in Assay buffer [50 mM NaH_2_PO_4 (_pH 7.4), 5 mg 3, 3’-Damiobenzidine tetra hydrochloride hydrate (DAB) (Sigma, St. Louis, USA) and 50 μM cytochrome C (Sigma, St. Louis, USA)].

### Immunoblotting

Proteins separated by BN-PAGE or SDS-PAGE were transferred to 0.22 μm PVDF membrane (Bio-Rad, Hercules, USA) using a semi-dry transfer system (15 V for 2 h, Bio-Rad, Hercules, USA). After blocking the membranes with 5% non-fat dried milk in Tris-buffered saline with Tween (TBST) [150 mM NaCl, 15 mM Tris-HCl (pH 7.5), 0.1% Tween 20] for approximately 2 h, proteins were probed using anti-NDUFA13 (1:1,000; MitoSciences, Eugene, USA) or anti-succinate dehydrogenase complex, subunit A (SDHA) (1:1,000; MitoSciences, Eugene, USA), anti-core2 (1:1,000; MitoSciences, Eugene, USA), anti-cytochrome c oxidase subunit IV (COXIV) (1:1,1000; MitoSciences, Eugene, USA), anti-ATP synthase subunit alpha (1:1,1000; MitoSciences, Eugene, USA), anti-voltage-dependent anion channel (VDAC) (1:1,000; Cell Signaling, Danvers, USA) antibodies, and then incubated with an alkaline phosphatase-conjugated anti-mouse IgG (1:2,000; Cell Signaling, Danvers, USA) or horseradish peroxidase-conjugated anti-rabbit IgG (1:2,000; Cell Signaling, Danvers, USA) secondary antibody. Membranes were washed with TBST after each antibody probing. Signals were detected using Super Signal West Pico Chemiluminescent Substrate (Thermo Scientific, Waltham, USA) or 5-bromo-4-chloro-3-indolyl-phosphate/nitro blue tetrazolium (BCIP/NBT) substrate (Promega, Madison, USA). Integrated optical density (IOD) quantification was performed using a Gel-Pro Analyzer 4.0 (MediaCybernetics, Warrendale, USA).

### Statistical analysis

The data are presented as mean ± SD of three independent experiments. Statistical significance was evaluated by a one-way ANOVA or independent Student’s *t* test using SPSS 16.0 software (IBM, Armonk, USA). A null hypothesis was rejected when *P* <0.05.

## Additional Information

**How to cite this article**: Fang, H. *et al.* Exercise intolerance and developmental delay associated with a novel mitochondrial ND5 mutation. *Sci. Rep.* **5**, 10480; doi: 10.1038/srep10480 (2015).

## Supplementary Material

Supplementary Information

## Figures and Tables

**Table 1:** Respiratory chain enzyme activities.

		**Reference Value (%)**			
	**Activity^a^ (%)**	**Deficiency**	**Low**	**Normal**	**Result**
Complex I	57.2	<64.5	64.5 ~ 76.3	76.3 ~ 161.3	Deficiency
Complex II	139.4	<62.5	62.5 ~ 74.1	74.1 ~ 174.1	Normal
Complex I + III	70.5	<63.0	63.0 ~ 73.1	73.1 ~ 177.8	Low
Complex IV	55.3	<62.3	62.3 ~ 71.9	71.9 ~ 238.0	Deficiency
Complex V	79.9	<60.7	60.7 ~ 75.2	75.2 ~ 566.2	Normal

^a^activity was presented as ratio of ( complex enzyme activity/citrate synthase).

**Table 2:** Analysis of whole mitochondrial genome.

**Position**	**Gene**	**rCRS base**	**Mutation**	**AA change**	mtDNA databases*
249	D-loop	A	del	no	Polymorphic Sites
263	D-loop	A	G	no	Polymorphic Sites
523	D-loop	A	del	no	Polymorphic Sites
524	D-loop	C	del	no	Polymorphic Sites
16129	D-loop	G	A	no	Polymorphic Sites
16172	D-loop	T	C	no	Polymorphic Sites
16304	D-loop	T	C	no	Polymorphic Sites
16519	D-loop	T	C	no	Polymorphic Sites
750	12s rRNA	A	G	no	Polymorphic Sites
1438	12s rRNA	A	G	no	Polymorphic Sites
1694	16s rRNA	T	C	no	Polymorphic Sites
2706	16s rRNA	A	G	no	Polymorphic Sites
3107	16s rRNA	C	del	no	Polymorphic Sites
4086	ND1	C	T	no	Polymorphic Sites
3970	ND1	C	T	no	Polymorphic Sites
4769	ND2	A	G	no	Polymorphic Sites
8860	ATPase6	A	G	Thr -> Ala	Polymorphic Sites
9055	ATPase6	G	A	Ala -> Thr	Polymorphic Sites
10310	ND3	G	A	no	Polymorphic Sites
11719	ND4	G	A	no	Polymorphic Sites
13928	ND5	G	C	Ser -> Thr	Polymorphic Sites
12882	ND5	C	T	no	Polymorphic Sites
**12955**	**ND5**	**A**	**G**	**Asn ->Asp**	**Not Reported**
13759	ND5	G	A	Ala -> Thr	Polymorphic Sites
14766	Cytb	C	T	no	Polymorphic Sites
15326	Cytb	A	G	Thr -> Ala	Polymorphic Sites
6392	cox 1	T	C	no	Polymorphic Sites
6962	cox 1	G	A	no	Polymorphic Sites
7028	cox1	C	T	no	Polymorphic Sites

del: deletion;* databases: MITOMAP, mtDB and mtSNP.
