# Supplementary material for: Exercise intolerance and developmental delay associated with a novel mitochondrial ND5 mutation
Source: Sci Rep. 2015 May 27;5:10480. doi: 10.1038/srep10480 (PMC4444849; doi:10.1038/srep10480)
Supplement: Supplementary Information [file srep10480-s1.doc]

**Exercise intolerance and developmental delay associated with a novel mitochondrial ND5 mutation**

Hezhi Fang^1†^*, Hao Shi^1†^, Xiyuan Li^2†^, Dayan Sun^1^, Fengjie Li^1^, Bin Li^1^, Yuan Ding^2^, Yanyan Ma^2^, Yupeng Liu^2^, Yao Zhang^2^, Lijun Shen^1^, Yidong Bai^3^, Yanling Yang^2^*, Jianxin Lu^1^*

^1^ Key Laboratory of Laboratory Medicine, Ministry of Education, Zhejiang Provincial Key Laboratory of Medical Genetics, College of Laboratory Medicine and Life sciences, Wenzhou Medical University, Wenzhou 325035, Zhejiang, China;

^2^ Department of Pediatrics, Peking University First Hospital, Beijing 100034, China;

^3^ Department of Cellular and Structural Biology, University of Texas Health Science Center at San Antonio, San Antonio, TX 78229, USA

***** Correspondence and requests for materials should be addressed to L.J ([jxlu313@163.com](mailto:jxlu313@163.com)), or Y.Y ([organic.acid@126.com](mailto:organic.acid@126.com)) F.H ([hezhifang990909@gmail.com](mailto:hezhifang990909@gmail.com)).

**^†^** These authors contributed equally to this work.

#17 #9 #18

#17 #9 #18


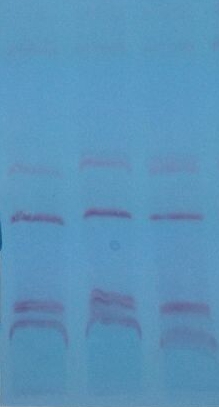


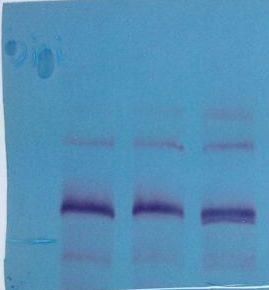


Complex I

#17 #9 #18

Complex IV


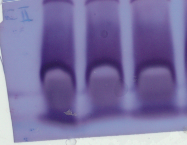


Complex II

#17 #9 #18

#17 #9 #18


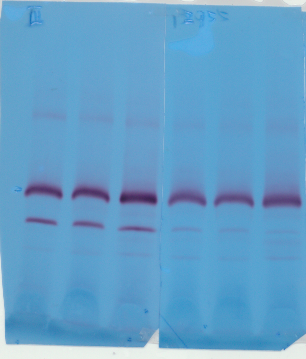


Complex V

Complex III


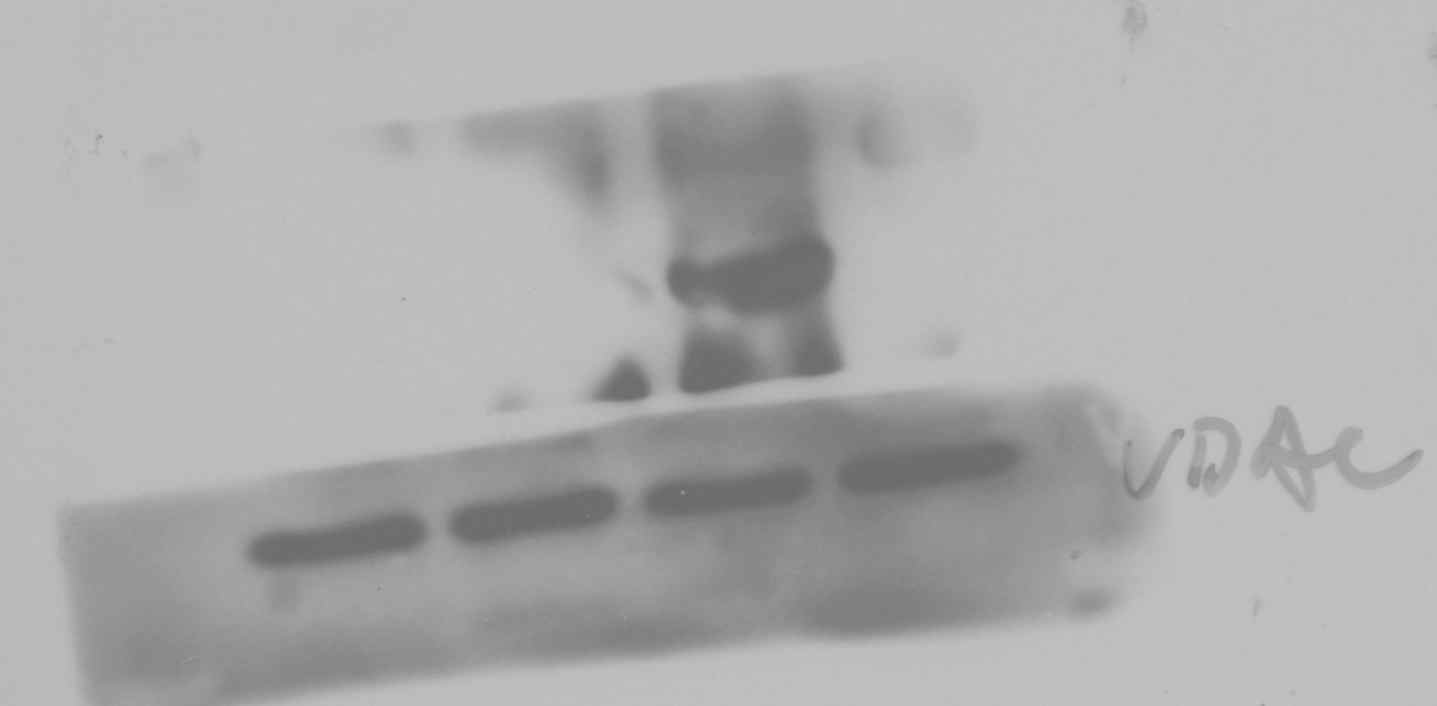


#17 #9 #18

#17 #9 #18

VDAC

#17 #9 #18

**Supplementary Figure 1 (for Figure 2A)**

**m.12955A>G impaired respiratory complex assembly**


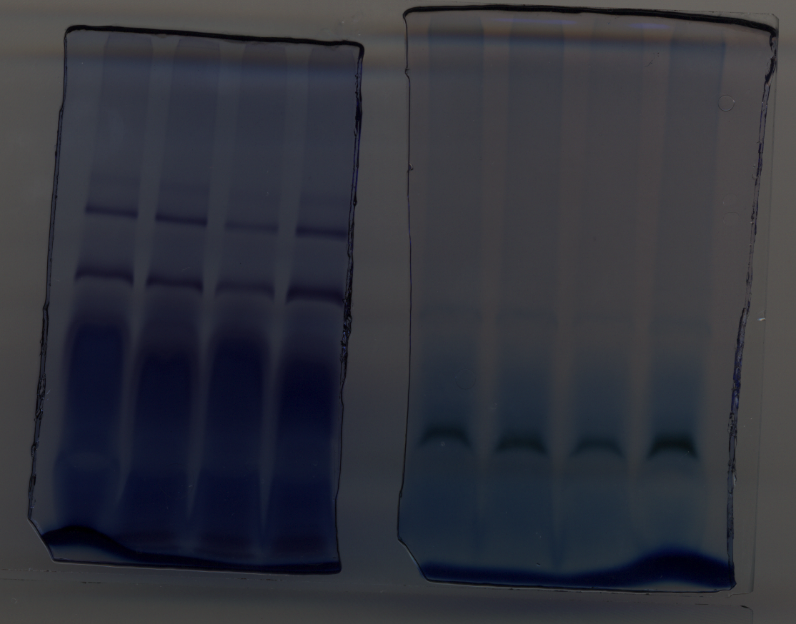

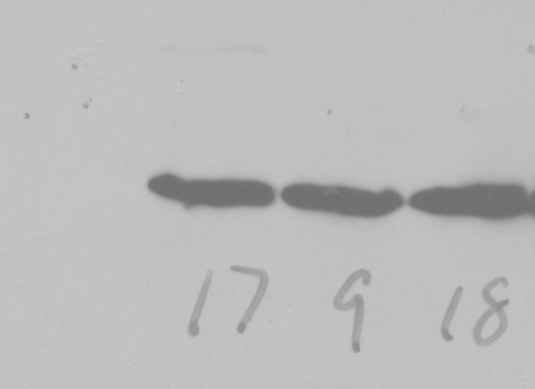


#17 #9 #18

#17 #9 #18

Complex I

Complex IV

VDAC

**Supplementary Figure 2 (for Figure 2B)**

**m.12955A>G impaired the activity of respiratory complex I and IV**

**Supplementary Figure 3 (for Figure 4). Enzyme activity assay of mitochondrial respiratory complex I (NADH:ubiquinone oxidoreductase)**

Enzyme activity of mitochondrial complex I was determined and normalized with citrate synthase activity in clone #9 and #17 cells.

Error bars, ± SD. *, *P* < 0.05;

**
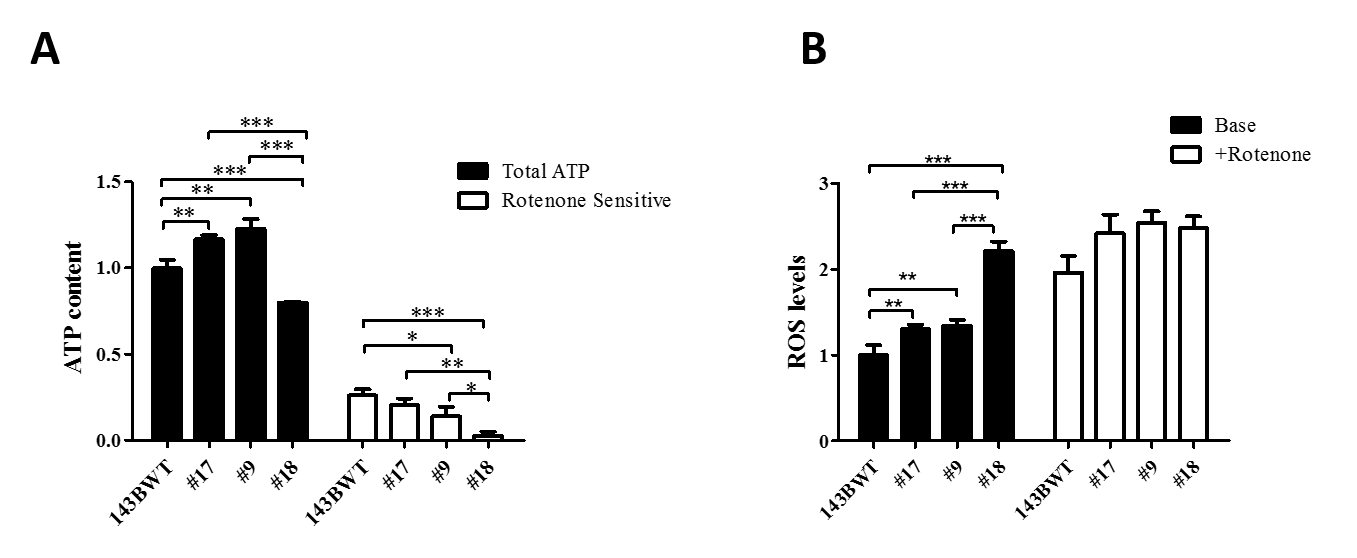
**

**Supplementary Figure 4 (for Figure 4). Mitochondrial function and cytotoxicity**

(A) Total ATP content was measured in #17, #9, #18 and143B wild type (WT) cells; the rotenone-resistant ATP content was determined in cells treated with 200 nM rotenone for 24 h. The rotenone-sensitive (RS) ATP content was calculated by subtracting the rotenone-resistant component from the total ATP content (n ≥ 4). **(**B) Mitochondrial superoxide levels (ROS) were determined in #17, #9, #18 and143B WT cells; complex I dependent ROS levels were measured in cells exposed to 1µM rotenone for 1 h (n ≥ 4).The values for ATP, lactate and ROS were normalized by protein concentration. Error bars, ±SD. *, *P* < 0.01; **, *P* < 0.001.
